# Risk factors for the leakage of the repair of duodenal wounds: a secondary analysis of the Panamerican Trauma Society multicenter retrospective review

**DOI:** 10.1186/s13017-023-00494-8

**Published:** 2023-04-04

**Authors:** Alberto García, Alvaro I. Sanchez, Paula Ferrada, Luke Wolfe, Juan Duchesne, Gustavo P. Fraga, Elizabeth Benjamin, Andre Campbell, Carlos Morales, Bruno M. Pereira, Marcelo Ribeiro, Martha Quiodettis, Gregory Peck, Juan C. Salamea, Vitor F. Kruger, Rao Ivatury, Thomas Scalea

**Affiliations:** 1 Division of Trauma and Acute Care Surgery, Department of Surgery, Department of Intensive Care, Fundación Valle del Lili, Cra 98 No. 18–49, 760032 Cali, Colombia; 2 Division of Trauma and Acute Care Surgery, Department of Surgery, Universidad del Valle, Cali, Colombia; 3 Department of Surgery, Universidad Icesi, Cali, Colombia; 4 Division of Thoracic Surgery, Department of Surgery, Fundación Valle del Lili, Cali, Colombia; 5 Division of Trauma and Acute Care Surgery, Innova Fairfax Hospital, Falls Church, VA USA; 6 Department of Surgery, Virginia Commonwealth University, Richmond, VA USA; 7 Department of Surgery, Tulane University, LA New Orleans, USA; 8 Department of Trauma and Acute Care Surgery, University of Campinas, Campinas, Brazil; 9 Department of Surgery, Grady Memorial Hospital, Atlanta, GA USA; 10 Department of Surgery, University of California, San Francisco, CA USA; 11 Department of Surgery, Universidad de Antioquia, Medellín, Colombia; 12 Consultant General and Trauma Surgeon, Chair Division of Trauma, Burns, Critical Care and Acute Care Surgery, Sheikh Shakhbout Medical City Mayo Clinic, Abu Dhabi, United Arab Emirates; 13 Division of Trauma and Acute Care Surgery, Hospital Santo Tomas, Panama City, Panama; 14 Department of Surgery, Robert Wood Johnson Place, New Brunswick, NJ USA; 15 Department of Surgery, Hospital Vicente Corral Moscoso, Cuenca, Ecuador; 16 Department of Surgery, Shock Trauma Center, University of Maryland, MD College Park, USA; 17 University of Vassouras, Rio De Janeiro, Brazil; 18 Santa Casa de Campinas, Campinas, Brazil

## Introduction

Duodenal injury (DI) is infrequent. It has been reported in 0.003% to 0.5% of trauma admissions [1–3] and has been found in 3.1% to 5% trauma laparotomies [2, 4].

Most of the mortality occurs early and is related to associated lesions. Late deaths are associated with infections and multiple organ failure.

Among late morbidity, duodenal leakage (DL) and fistula have been reported in a wide range from 0 to 37.5% [5, 6], with a median of 6.1%. They are associated with higher rates of intraabdominal abscesses, prolongation of the stay in the ICU and the hospital and higher mortality [7–9].

Complex techniques, such as diverticulization [10, 11], pyloric exclusion (PE) [12], decompressive tube duodenostomy [13], were devised to prevent the exposition of the duodenal repair to saliva and gastric secretion, to reduce the pressure in the duodenal lumen or both, and as a consequence the risk and the impact of DL. They have been progressively abandoned in favor of primary repair, as in the last three decades they failed to show better outcomes.

Several authors have investigated the risk factors for DL. Still most of the evidence comes from retrospective series and lacks enough sample size, a precise definition of the studied morbidity and bivariate analyses, which precludes to know the influence of potential confounders [2, 3, 8, 9, 14–17]. Identified risk factors include shock and trauma severity. The associated pancreatic injury seems to increase the risk of DL.

Because of the mentioned limitations, the contribution of the complex techniques to reduce or increase the risk of DL has not been clarified.

A recently published multicentric study from the Panamerican Trauma Society (PTS), which had enough power, suggested that primary repair is safe in most duodenal injuries [18].

We performed a secondary analysis of the PTS database to evaluate the impact of the leakage of duodenal injuries surgically treated and to know the risk factors for DL, including the type of surgical repair.

## Materials and methods

A retrospective multicenter trial was conducted, including patients from 11 PTS centers.

Recruitment methods, collection of the information, and ethical considerations were previously reported [18].

Subjects 18 years and older with duodenal injuries, surgically treated from 2006 to 2017, were included. Patients who died in the first 48 h after the trauma and subjects without classification of the duodenal lesion severity or cases in which the outcome was not registered were excluded.

Demographics, trauma mechanism, shock on admission, injury severity, associated injuries, transfusions, and type of repair were examined as potential risk factors for a leak of the duodenal repair.

The severity of the duodenal injuries was classified according to the American Association for the Surgery of Trauma (AAST) severity scale. Grade 3 wounds were categorized independently for the analysis because they exhibited a higher risk of leakage, sepsis, and death.

The duodenal repairs were classified according to their relative risk of DL as "primary repair", "suture + duodenostomy", and "complex repairs". This category included PE and ligation with reconstruction or a Whipple's procedure in a subsequent procedure.

The analysis was performed with STATA 15.1^®^ (College Station TX). Categorical variables are presented as quantities and proportions—continuous variables as mean and standard deviation (SD) or median and interquartile range (IQR), after normality analysis.

Comparisons were made between patients who developed DL and patients who did not.

Proportions were compared with Chi^2^ or Fisher's exact test, as indicated. Continuous variables were compared with Student's test or Wilcoxon–Mann–Whitney test, according to normality.

Models were developed to identify predictors of duodenal leakage and sepsis. Potential predictors of DL were analyzed with simple logistic regressions. Variables with a *p* < 0.1, including the categorized duodenal repair, were included in a multiple logistic regression. The final models were evaluated with ROC curves and Hosmer–Lemeshow goodness-of-fit test.

## Results

A total of 378 patients were registered. Ninety of them met one or more exclusion criteria, being the most frequent exclusion causes death during the first 48 h after trauma (*n* = 61), and age < 18 years old (*n* = 30). The remaining 288 were selected for the analysis.

Median age was 29 years (IQR 22–43), and 236 (81.9%) of the subjects were males. Penetrating trauma occurred in 223 (77.3%). Forty-seven patients (16.3%) were hypotensive at admission, and 126 (43.8%) received transfusions before surgery. (Table 1).

**Table 1:** Descriptive statistics. Comparison by the leak of the duodenal repair

	Total (*n* = 288)	No leak (*n* = 230)	Leak (*n* = 50)	*p*-value
Age (years), median (IQR)	29 (22–43)	30 (22–43)	26.5 (22–37)	0.177**
Sex				
Males, n (%)	236 (81.9)	194 (81.5)	42 (84.0)	0.840*
Females, *n* (%)	52 (18.1)	44 (18.5)	8 (16.0)	
Injury mechanism				
Penetrating, n (%)	223 (77.3)	181 (76.1)	42 (84.0)	0.477*
Blunt, n (%)	65 (22.6)	57 (23.9)	8 (16.0)	
SBP in the ER (mm Hg), median (IQR)	111.5 (91.5–130)	116 (96–131)	100 (80–120)	< 0.001**
Hypotension at arrival to the ER, n (%)	47 (16.3)	31 (13.0)	16 (32.05)	< 0.001*
Transfusion before first surgery, n (%)	126 (43.8)	100 (42.0)	26 (52.0)	0.212*
PRBC transfused (units), median (IQR)	2 (0–5)	1 (0–5)	2 (0–5)	0.277**
Massive transfusion, n (%)	64 (22.2)	51 (21.4)	13 (26.0)	0.460*
ISS, median (IQR)	20 (16–26)	18 (16–25)	25 (17–26)	0.011**
Abdominal AIS, median (IQR)	3 (3–4)	3 (2–4)	4 (3–4)	< 0.001**
Duodenal AAST grade, median (IQR)	3 (3–3)	3 (2–3)	3 (3–3)	0.248**
Duodenal AAST grade 3, n (%)	180 (62.5)	139 (58.4)	41 (82.0)	0.002*
Associated intraabdominal injuries				
None, n (%)	28 (9.7)	24 (10.1)	4 (8.0)	0.797*
Liver, n (%)	119 (41.3)	95 (39.9)	24 (48.0)	0.344*
Colon, n (%)	102 (35.4)	84 (35.3)	18 (36.0)	1.000*
Pancreas, n (%)	83 (28.8)	56 (23.5)	27 (54.0)	< 0.001
Stomach, n (%)	67 (23.3)	50 (21.0)	17 (34.0)	0.064*
Major vascular, n (%)	59 (20.5)	51 (21.4)	8 (16.0)	0.446*
Small bowel, n (%)	47 (16.6)	40 (16.8)	7 (14.0)	0.833*
Kidney, n (%)	59 (20.5)	46 (19.3)	13 (26.0)	0.335*
Spleen, n (%)	22 (7.6)	18 (7.6)	4 (8.0)	1.000*
Surgical treatment				
Primary repair, n (%)	227 (78.8)	201 (84.5)	26 (52.0)	< 0.001*
Suture + duodenostomy, n (%)	27 (9.4)	19 (8.0)	8 (16.0)	
Complex repairs, n (%)†	34 (11.8)	18 (7.6)	16 (32.0)	

*SBP* Systolic blood pressure, *ER* Emergency room, *PRBC* Packed red blood cells, *IQR* Interquartile range, *AIS* Abbreviated Injury Scale, *AST* American Association for the Surgery of Trauma, *ISS* Injury Severity Score

*Fisher’s exact test

**Wilcoxon–Mann–Whitney test

†Pyloric exclusion, diverticulization, others

One hundred and eight patients (38.0%) had extraabdominal injuries. This proportion was higher among blunt trauma patients (56.3% vs. 32.7%). Median (IQR) ISS was 20 (16–26) (Table 1).

The AAST duodenal injury severity grade was 3 in 180 cases (62.5%) and 4 or 5 in 40 (13.9%) (Table 2). Median (IQR) of abdominal AIS was 3 (3–4) (Table 1).

**Table 2:** Trauma characteristics and outcomes according to duodenal trauma severity

Variable	AAST Duodenal injury grade			
	1 and 2	3	4 and 5	*p*-value
Number of patients (%)	68 (23.6)	180 (62.5)	40 (13.9)	–
Age (years), median (IQR)	29.5 (22–43)	29 (22–40)	28.5 (21–40.5)	0.935**
SBP in the ER (mm Hg), median (IQR)	112 (99–125)	110 (90–130)	120 (100–138)	0.140**
Hypotension at arrival to the ER, n (%)	11 (16.2)	31 (17.2)	5 (12.5)	0.845*
Transfusion before first surgery, n (%)	23 (33.8)	91 (50.6)	12 (30.0)	0.10*
PRBC transfused (units), median (IQR)	1 (0–4)	2 (0–6)	0 (0–3.5)	0.231**
Massive transfusion, n (%)	15 (22.1)	41 (22.8)	8 (20.0)	0.978*
ISS, median (IQR)	18 (15–25)	21 (16–26)	16 (10.5–25)	0.005**
Abdominal AIS, median (IQR)	3 (2–4)	3 (3–4)	3.5 (3–5)	0.031**
ICU admission, n (%)	38 (55.9)	134 (74.4)	29 (72.5)	0.019*
Hospital LOS, n (%)	13 (8–25)	14.5 (9–31)	18 (10.5–44.5)	0.089**
Leak of the duodenal repair, n (%)	5 (7.4)	41 (22.8)	4 (10.0)	0.006*
Need for unplanned surgery, n (%)	17 (25.0)	69 (38.3)	17 (42.5)	0.094*
Sepsis, n (%)	10 (14.7)	47 (26.1)	8 (20)	0.165*
Mortality, n (%)	0 (0)	31 (17.2)	5 (12.5)	< 0.001*

*AAST* American Association for the Surgery of Trauma, *SBP* Systolic blood pressure, *ER* Emergency room, *PRBC* Packed red blood cells, *IQR* Interquartile range, *ISS* Injury Severity Score, *AIS* Abbreviated Injury Scale

*Fisher’s exact test

**Kruskal–Wallis test

The most frequent intraabdominal injured organ was the liver in 119 cases (41.3%), followed by the colon in 102 (35.4%), and the pancreas in 83 (28.8%). Fifty-nine (20.5%) patients had an abdominal vascular injury. In 28 cases (9.7%), there was not an abdominal associated injury (Table 1).

DL developed in 50 subjects (17.4%). Compared to those without leak, patients with leakage had significantly lower SBP at admission (100 mm Hg, IQR 80–120, vs.116 mm Hg, IQR 96–131), higher ISS (25, IQR 17–26, vs.18, IQR 16–25), higher abdominal AIS (4, IQR 3–4, vs.3, IQR 2–4), and a higher proportion of AAST grade 3 DI (82.0% vs. 58.4%). Pancreatic injury was most frequent in this group (54.0% vs. 23.5%) (Table 1).

The duodenal injury was treated most frequently by primary repair (78.8%). In 27 (9.4%) cases, a repair plus a descompressive duodenostomy was performed, in 26 (9.0%) a PE, with or without gastro-jejunostomy, and in 5, other methods of reconstruction. For the purpose of the analysis, PE and other methods were grouped as “complex repairs” due to their similar leak rate.

**Fig. 1:**
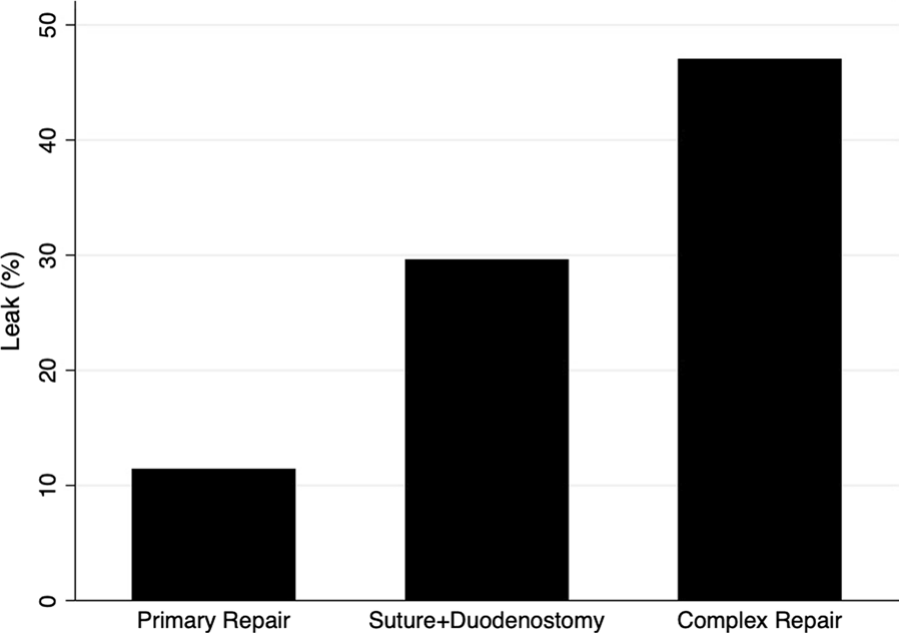
Type of repair and risk of duodenal repair leakage

Compared with primary repair, patients managed with suture + duodenostomy or complex repairs leaked more frequently (Fig. 1).

Table 2 shows the comparison between grade 3 and the other grades of AAST DI. Grade 3 patients had more severe systemic trauma and associated abdominal injury, leaked, and developed sepsis more frequently. Mortality among them was higher.

### Predictors of leak of the duodenal repair

Age, hypotension, ISS, abdominal AIS, duodenal AAST grade 3, associated injury of the pancreas and the liver, and the type of duodenal repair were identified as potential risk factors for DL in the univariate analysis (Table 3).

**Table 3:** Analysis of risk factors for leak of duodenal repair

Variable	Univariable analysis		Multivariable analysis	
	OR (95% C.I.)	*p*-value	OR (95% C.I.)	*p*-value
Age, median (IQR)	0 .963 (0.951–1.002)	0.077	–	0.401
Penetrating injury mechanism	1.653 (0.734–3.726)	0.225	–	0.839
Hypotension at arrival	3.142 (1.554–6.353)	0.001	3.386 (1.516–7.565)	0.003
ISS	1.034 (1.001–1.068)	0.043	–	0.623
Abdominal AIS	1.908 (1.362–2.672)	< 0.001	1.967 (1.331–2.908)	0.001
Duodenal AAST grade	1.262 (0.815–1.954)	0.298	–	
Duodenal AAST grade 3	3.245 (1.508–6.981)	0.003	3.367 (1.467–7.728)	0.004
*Associated intraabdominal injuries*				
Liver	1.389 (0.753—2.563)	0.293	–	
Pancreas	3.815 (2.029–7.175)	< 0.001	–	0.166
Major vascular	0.698 (0.309–1.581)	0.389	–	
Stomach	1.937 (0.998–3.759)	0.051	–	0.386
*Surgical treatment*				
Primary repair (reference)	1		1	
Suture + duodenostomy	3.255 (1.295–8.180)	0.012	5.343 (1.829–15.605)	0.002
Complex repairs	6.872 (3.126–15.105)	< 0.001	6.941 (2.905–16.588)	< 0.001

Area under ROC curve = 0.824 (0.766–0.883)

Goodness of fit *p* = 0.271

The MLR identified as independent predictors of leakage of the repair of the duodenal lesion hypotension on admission, O.R. (IQR) 3.386 (1.516–7.565), abdominal AIS, 1.967 (1.331–2.908) for each AIS point, duodenal AAST grade 3, 3.367 (1.467–2.908), and the duodenal repair with techniques different from primary repair, [O.R. (IQR) 5.343 (1.829–15.605) for primary suture + duodenostomy and 6.941 (2.905–16.558) for other complex repairs].

The created model had a good discriminative ability of the risk of DL (AUROC = 0.824 (0.766–0.883), and sufficient goodness to fit (*p* = 0.271).

### Outcomes associated with the leak of the duodenal repair.

Compared with the group with no leak, the patients who leaked were admitted more frequently to the ICU (84.0% vs. 66.6%). The ICU LOS was more prolonged among the leak group [median (IQR) 21 (10–31), vs. 5.5 (3–12) days]. Additionally, patients who leaked spent more time in the hospital [median (IQR) 32 (14–52), vs. 13 (8.5–22) days].

The subjects with a leakage required more frequently unplanned surgeries, intraabdominal abscess drainage, and mechanical ventilation (Table 4).

**Table 4:** Duodenal trauma. Outcomes compared by the leak of the duodenal repair

Variable	Total (*n* = 288)	No leak (*n* = 238)	Leak (*n* = 50)	P value
ICU admission, n (%)	201 (69.8)	159 (66.6)	42 (84.0)	0.017*
ICU LOS† days, median (IQR)	7 (4–16)	5.5 (3–12)	21 (10–31)	< 0.001**
Hospital LOS days, median (IQR)	15 (9–30)	13 (8.5–22)	32 (14–52)	< 0.001**
Sepsis, n (%)	65 (22.6)	34 (14.3)	31 (62.0)	< 0.001*
Intraabdominal abscess, n (%)	30 (10.4)	20 (8.4)	10 (20.0)	0.022*
Need for unplanned surgery, n (%)	103 (35.8)	79 (33.2)	24 (48.0)	0.053*
Mechanical ventilation, n (%)	82 (28.5)	57 (24.0)	25 (50.0)	< 0.001*
Renal replacement therapy, n (%)	22 (7.6)	16 (6.7)	6 (12.0)	0.238*
Hospital readmission, n (%)	41 (14.2)	35 (14.7)	6 (12.0)	0.824*
Mortality, n (%)	36 (12.5)	26 (10.9)	10 (20.0)	0.098*

*ICU Intensive Care Unit LOS Length of Stay*

*Fisher’s Exact Test

**Wilcoxon–Mann–Whitney test

^†^For the patients admitted to the ICU

There were non-statistically significant increases in the need for renal replacement therapy and mortality. The readmission rate was similar in both groups (Table 4).

Multiple logistic regression identified DL as an independent risk factor for sepsis, along with hypotension, ISS, massive transfusion, and the use of complex procedures for repairing the DI (Table 5).

**Table 5:** Risk factors for sepsis after duodenal trauma

Variable	Univariable analysis		Multivariable analysis	
	OR (95% C.I.)	*p*-value	OR (95% C.I.)	*p*-value
Age, median (IQR)	0 .988 (0.967–1.001)	0.280	–	0.466
Penetrating injury mechanism	2.445 (1.101–5.438)	0.028	–	0.141
Hypotension at arrival	3.616 (1.866–7.007)	< 0.001	2.218 (1.003–4.905)	0.049
Massive transfusion	2.949 (1.606–5.413)	< 0.001	2.553 (1.246–5.231)	0.010
ISS (every 10 points)	1.802 (1.322–2.456)	< 0.001	1.651 (1.144–2.384)	0.007
Abdominal AIS	1.505 (1.122–2.017)	0.002	–	0.799
Duodenal AAST grade	1.196 (0.806–1.776)	0.375	–	–
Duodenal AAST grade 3	1.767 (0.994–3.238)	0.065	–	0.988
Associated intraabdominal injuries				
Stomach	1.664 (0.898–3.085)	0.106	–	0.582
Pancreas	3.568 (1.999–6.368)	< 0.001	–	0.206
Kidney	2.324 (1.242–4.346)	0.008	–	0.277
Leak of the duodenal repair	7.083 (3.341–15.012)	< 0.001	7.083 (3.341–15.012)	< 0.001
Complex repair of the duodenum*	4.367 (2.357–8.055)	< 0.001	2.937 (1.425–6.051)	0.003

*Area under ROC curve* = *0.819 (0.758*–*0.879)*

*Goodness of fit p* = *0.546*

*IQR* Interquartile range, *ISS* Injury Severity Score, *AAST* The American Association for the Surgery of Trauma

*Duodenal suture + duodenostomy or pyloric exclusion or diverticulizaction or other complex repairs

## Discussion

Leakage of the repair of a duodenal lesion with or without fistula formation is one of the most feared complications in the surgical treatment of duodenal trauma, with a median of 6.3% in the published series [1, 6, 8, 9, 12–15, 17–33]. It has been associated with a higher risk of intraabdominal infection [8, 15], the need for support [8, 15, 29], prolonged stay [8, 15, 29], and a higher death risk [1, 8, 9, 13, 15, 20, 21, 24, 25, 27, 29].

In the PTS cohort, we identified leakages in 17.4% of the cases, which showed association with a higher risk of intraabdominal abscess, sepsis, ICU admission, and ventilatory support. ICU and hospital stay were longer.

The multivariate analysis of the sepsis risk factors revealed that DL contributes independently of trauma severity, shock, massive transfusions, and the technique used to repair the duodenal injury.

The probability of death was 1.8 times higher in the subjects with leakage. This difference did not reach statistical significance. Except for Levison’s study [23], which reported a slightly lower mortality rate in the group of the patients who leaked, the authors who analyzed this association found a higher risk of death in the leak subjects, with a median of 2.8 [1, 8, 9, 13, 15, 20, 21, 24, 25, 27, 29]. The intriguing Levinson's finding may be the consequence of survival bias. The author did not exclude the early deaths. Eight of the 17 patients who died did it intraoperatively by exsanguination. They did not have a chance to leak despite the severity of their trauma, modifying the result falsely.

The risk factors for DL have not been appropriately studied. Previous publications examined all duodenal complications, performed univariate analyses, or had low statistical power. In 1999, Timaran and coworkers studied 152 patients, 27 of them with duodenal complications. In a multivariate analysis, they found shock, ATI > 25, and the coexistence of colonic, pancreatic, or superior mesenteric vessels injury as independent risk factors [15]. In 2008, Fraga et al., in univariate analysis of duodenal and non-duodenal complications, occurring in 47 of 77 patients, identified association with altered RTS, ATI > 25, ISS > 25, and procedures different to primary repair [17]. In 2016, Schroeppel et al. compared subjects who leaked with individuals who did not. They did not identify significant differences in the compared variables [8]. In 2019, Weale published a similar comparison reporting a lower arterial PH, a higher lactic acid, and more frequent damage control surgeries in the patients who developed a duodenal leak [9].

Our study collected patients from 11 trauma centers from North, Central, and South America. It included an adequate number of subjects and outcomes to perform the statistical analysis required to identify the variables associated with the leak of the duodenal repair. We confirm the role of shock and trauma severity as risk factors for DL and evidence the risk associated with the more complex repairs, independently of the presence or the magnitude of the other factors.

Complex procedures were devised, to decompress the duodenum or to deviate the intestinal content from the repair, to prevent the fistula formation or to ameliorate its impact. Some of them, such as diverticulization, proved to be excessively aggressive or morbid. The merits of others, such as pyloric exclusion or duodenal decompression, are still debated.

Pyloric exclusion with gastro-jejunostomy, as described by Vaughan [12], or without it as proposed by Ginzburg [34] and Ferrada [35], has been the preferred method to treat duodenal injuries judged as severe.

One of the main difficulties in selecting candidates for a PE is the definition of severe duodenal trauma. Ben Taub Hospital [12, 22] and Denver Hospital [36] surgeons reported using PE in severe duodenal or pancreatoduodenal injuries without clearly defining severe trauma. Both groups reported PE in 41% of their cases. Nassoura et al., on the other hand, proposed ATI > 40 or duodenal injury score ≥ 4 as severity criteria. They performed PE in 3 out of 66 patients [14]. Additionally, the reports describing the surgical treatment according to trauma severity showed PE was used among severity grades 2 to 5, giving evidence of inconsistencies in the indication [18, 27, 36, 37].

The technique was created to reduce the risk of complications, which has not been proven. The publications from Houston containing the technique's description showed leakages only in the group treated by PE [12, 22].

Some studies have evaluated the impact of PE. Seamon and coworkers studied patients with penetrating DI OIS ≥ 2, who survived > 48 h. They compared 14 subjects with PE with 15 managed with PR. PE patients had a higher proportion of grade 4 injuries (21% vs. 0), suffered complications more frequently (71% vs. 33%), and had a more extended hospital stay (24.3 ± 19.7 vs. 13.5 ± 7.7 days). None of the differences reached statistical significance [6].

Velmahos et al. included 50 patients with OIS > 2 DI, 16 with PE. The proportion of cases with injuries in D1 and D2 and subjects with pancreatic trauma were higher in the PE group (79% vs. 42%, *p* = 0.02) and (63% vs. 24%, *p* = 0.02), respectively. DL, intraabdominal infections, and systemic complications occurred with similar frequencies [31].

Dubose and coworkers analyzed patients from the National Trauma Data Bank with DI grades 2 to 5 who survived more than 24 h. They compared 119 subjects primarily repaired with 28 patients treated with PE. The proportions of patients with ISS > 20, abdominal AIS > 3, and DI > 3 were higher in the PE group, without statistical significance. Adjusted morbidity, mortality, ICU stay, hospital stay, and hospital charges were similar [30].

Our data showed a fourfold increase in the risk of leakage when a PE was used. It cannot be attributed to the trauma severity. The association persisted after adjustment by the other identified risk factors.

Duodenal decompression with tubes comprises a heterogeneous set of intraluminal lines, including gastrostomy, duodenostomy, and proximal and distal jejunostomy. It was proposed by Stone et al. as an adjunct to reduce the pressure within the duodenal lumen without opening or resecting the stomach [38]. Original Stone's publication reported zero duodenal complications in 18 patients treated with this method [38]. Corley and coworkers informed 15% of duodenal complications in decompressed patients, compared with 26% in not decompressed subjects [1]. Stone and Fabian reported 302 cases of DI. Decompression was used in 78%. Duodenal complications occurred in 0.4% of the patients treated with decompression and in 19% of the cases treated without it [13].

Other authors reported a high frequency of use of decompression, without similar results. Snyder et al. complemented the duodenal repair with decompression techniques in 53% of their cases. Duodenal morbidity was more frequent among decompressed patients, 12% versus 8% [21]. Schroeppel and coworkers informed using decompression in 50% of their cases. Duodenal leakage happened in 10% when decompression was used and 2% when it was not [8].

In our report, DL was three times more frequent in the repair + duodenostomy. The association persisted and its strength increased after the multivariate analysis. It confirms the independent contribution and suggest a role in increasing the risk of DL.

Nassoura et al. proposed primary repair as the management technique for most penetrating DI. Duodenal fistula developed in 4% of the PR patients [14]. Some authors have documented an increase in PR use without a parallel increase in the complications [39, 40]. In most contemporary reports, Talving and Weale informed PR in 87% and 97% of their cases, respectively, with a low leakage rate [9, 29].

The available literature and our results identify trauma severity (systemic and local) as the main determinant of leakage after the repair of a duodenal injury [9, 15, 17, 28]. Complex procedures including diverticulization, pyloric exclusion, and tube duodenostomy have failed to reduce the risk of duodenal complications. In fact, as our analysis shows, they can contribute to increase the risk. Resecting, practicing incisions, and anastomoses or inserting tubes for decompression sum to the traumatic burden and the operation's length, which can increase the risk of infectious complications. There is enough evidence of the biological and clinical impact of the trauma from the injury and the surgery [41–44] and the additional risk derived from unnecessary procedures [45–48]. Our findings can be considered part of this evidence.

Our study has several limitations. First, the retrospective nature introduces the risk of information bias. It was mitigated by using clear and simple definitions. Second, the collected information covers 10 years, with possible changes in the diagnostic strategies, surgical procedures, and resuscitation principles. The available information did not let us analyze the influence of the trends over time on the risk factors or the outcomes. Third, duodenal trauma is infrequent. The exposition of each surgeon is limited, and as a consequence, the practices may be inconsistent. Despite this, the associations between the complex procedures and the duodenal complication were robust.

On the other hand, the investigation has some strengths which must be mentioned. First, patients from 11 high-volume trauma centers from North America, Central America, and South America were included. It makes our conclusions more generalizable. Second, the explored information and used definitions permitted us to analyze the most critical technical aspects. Third, the assembled cohort's sample size and the number of outcomes observed allowed the analyses we performed.

## Conclusion

This retrospective multicentric analysis included 288 patients from 11 North and Latin America trauma centers. Hypotension at arrival, abdominal AIS, duodenal OIS = 3, and complex surgical procedures were identified as independent risk factors for the leakage of the repair of the duodenal injuries. Our findings permit us to recommend abandoning complex surgical procedures, including duodenal tube decompression, in favor of primary repair.

## Data Availability

Yes.
